# Recent advances in the role of miRNAs in post-traumatic stress disorder and traumatic brain injury

**DOI:** 10.1038/s41380-023-02126-8

**Published:** 2023-06-20

**Authors:** Ziyu Zhu, Xuekang Huang, Mengran Du, Chenrui Wu, Jiayuanyuan Fu, Weilin Tan, Biying Wu, Jie Zhang, Z. B. Liao

**Affiliations:** Department of Neurosurgery, The First Affiliated Hospital of Chongqing Medical University, Chongqing, 400016 China

**Keywords:** Psychiatric disorders, Neuroscience, Molecular biology

## Abstract

Post-traumatic stress disorder (PTSD) is usually considered a psychiatric disorder upon emotional trauma. However, with the rising number of conflicts and traffic accidents around the world, the incidence of PTSD has skyrocketed along with traumatic brain injury (TBI), a complex neuropathological disease due to external physical force and is also the most common concurrent disease of PTSD. Recently, the overlap between PTSD and TBI is increasingly attracting attention, as it has the potential to stimulate the emergence of novel treatments for both conditions. Of note, treatments exploiting the microRNAs (miRNAs), a well-known class of small non-coding RNAs (ncRNAs), have rapidly gained momentum in many nervous system disorders, given the miRNAs’ multitudinous and key regulatory role in various biological processes, including neural development and normal functioning of the nervous system. Currently, a wealth of studies has elucidated the similarities of PTSD and TBI in pathophysiology and symptoms; however, there is a dearth of discussion with respect to miRNAs in both PTSD and TBI. In this review, we summarize the recent available studies of miRNAs in PTSD and TBI and discuss and highlight promising miRNAs therapeutics for both conditions in the future.

## Introduction

Post-traumatic stress disorder (PTSD) and traumatic brain injury (TBI) are both prevalent and debilitating diseases afflicting millions of people around the world [1, 2]. PTSD is defined as a constellation of emotional and behavioral changes in response to traumatic or stressful events by the Diagnostic and Statistical Manuals of Mental Disorders (DSM-5) and is characterized by four symptom clusters: re-experiencing, avoidance, alterations in cognitions and mood, and arousal and hyperexcitation symptoms [3, 4]. TBI is defined as an alteration in brain function or other evidence of pathology caused by a biomechanical force [5]. It is not surprising that PTSD and TBI are commonly comorbid because the patients often sustain brain injuries during traumatic events, such as traffic accidents, violence, and natural disasters [6]. Importantly, TBI itself is a well-documented risk factor for PTSD development (especially mild TBI, while severe TBI is postulated to be protective against PTSD due to memory and conscious loss) [7]. On the other hand, PTSD might be a mediator of TBI pathological progression as well [8]. These intriguing connections between PTSD and TBI warrant further research and might lead to vigorous treatments for both diseases. microRNAs (miRNAs) are undoubtedly of widespread interest across many kinds of neurological diseases, and efforts have already been made to push miRNAs-based treatments into clinical application. For the past few years, a plethora of studies have been performed in search of possible therapeutic miRNAs towards PTSD or TBI. In this review, we focus on the recent findings from miRNAs research in PTSD and TBI and their implications for future treatments. A detailed discussion of pathophysiology and symptomology is outside the scope of the present review and can be found elsewhere [9].

## Background of miRNAs

miRNAs are small, non-coding RNAs that have been exhaustively studied given their important post-transcriptional regulation of gene expression. Functional studies have indicated that miRNAs participate in almost every cellular process that has been investigated so far and are associated with many pathological processes [10].

**Fig. 1:**
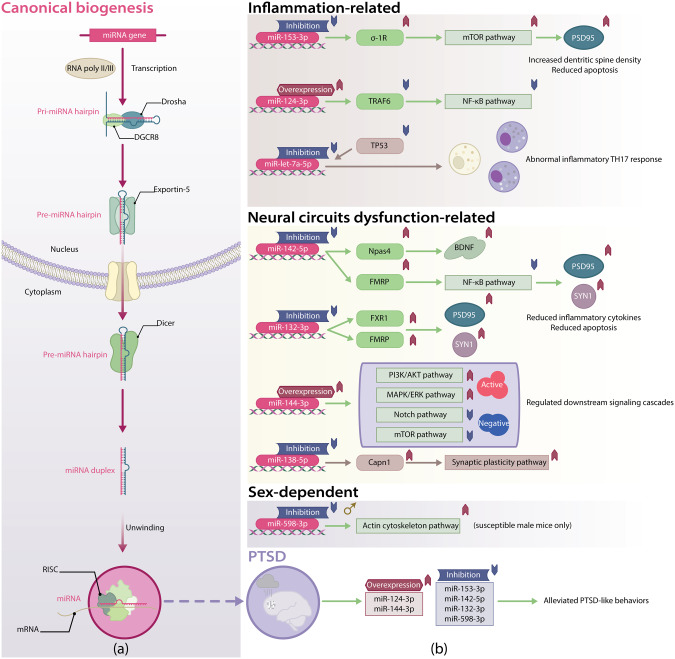
Canonical miRNA biogenesis and PTSD-TBI-common miRNAs reported in PTSD. **a** Canonically, after transcription by the RNA polymerase II or III, pri-miRNA hairpin undergoes nuclear processing by Drosha and DGCR8 and is next transported to the cytoplasm via Exportin-5 as pre-miRNA hairpin. The miRNA duplex is then formed through the Dicer processing. Finally, after unwinding the miRNA duplex, the guide strand is corporated into the RISC and guides it to the target mRNAs. **b** The eight PTSD-TBI-common miRNAs play different roles in PTSD. Overexpression of miR-144-3p and miR-124-3p improves PTSD symptoms; inhibition of miR-153-3p, miR-142-5p, miR-132-3p, and miR-598-3p is also favorable. However, inhibition of miR-138-5p and miR-let-7a-5p is involved in the pathological processes of PTSD.

The biogenesis of miRNA is a multistage process (See Fig. 1a). Canonically, the primary miRNA (pri-miRNA) hairpin is first transcribed by the RNA polymerase II or III. The pri-miRNA hairpin is then processed by the RNase III endonuclease Drosha and the RNA binding protein DiGeorge syndrome critical region 8 (DGCR8) to form the smaller precursor miRNA (pre-miRNA) hairpin. After transportation to the cytoplasm by Exportin-5, another RNase III endonuclease Dicer next digests pre-miRNA hairpin to miRNA duplex (guidance strand and passenger strand) [11–13].

Usually, the strand with lower 5’ end stability (lower C-G pairs) is more likely to be deemed as the guidance strand. The guidance strand is much more abundant and biologically functional than the passenger strand, which is non-functional and will be degraded [14]. Only the mature miRNA or the guidance strand is incorporated in the RNA-induced silencing complex (RISC) and serves as a negative regulator of gene expression. The mature miRNA guides RISC to targeted mRNA by recognizing the specific 3’ untranslated region (UTR), and consequently inhibits the protein synthesis through translation repression or de-adenylation of the mRNA [12, 15].

Nevertheless, growing evidence suggests that passenger strand can also be biologically active, and miRNAs are furthermore capable of interacting with proteins and DNAs, not only restricted to mRNAs [16]. Even more striking, contrary to the widely-accepted negative regulatory role, studies have found that nuclear activating miRNAs (NamiRNAs) could positively regulate gene expressions by enhancing the enhancer activation or competitively binding repressor proteins [17, 18]. Clearly, more work is required to fully illuminate the regulatory role of miRNAs involved in various biological processes.

## Search strategy

**Fig. 2:**
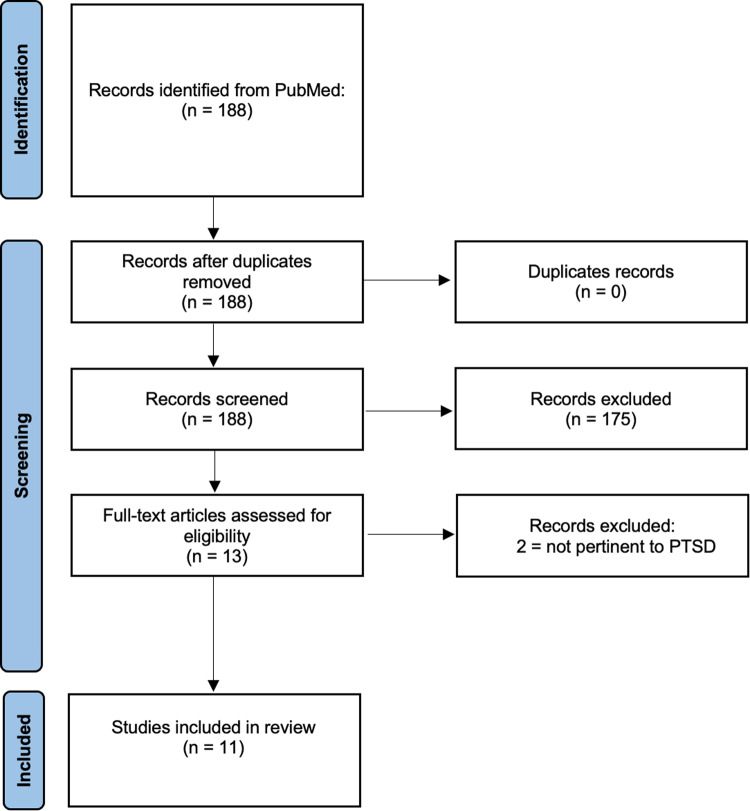
PRISMA diagram of the literature search process, including identification, screening, and inclusion.

The PubMed database was searched following the PRISMA guidelines using MeSH terms, including “Stress Disorders, Post-Traumatic”, “RNA, Untranslated”, “MicroRNAs”, “RNA, Circular”, and “RNA, Long Noncoding”, with corresponding entry terms. A total of 188 records were obtained and 11 records were finally included in this review. (See Fig. 2)

## Advances in miRNAs-based treatments in various neurological diseases

miRNAs are abundant in the central nervous system (CNS) and play a pivotal role in the pathogenesis of diverse diseases, such as cancer, epilepsy, PTSD, and TBI. Among these diseases, the most prominent application of miRNAs treatments is probably in cancer. Targeting multiple oncogenic genes, pathways, and miRNAs, miRNAs-based drugs showed favorable effects on survival in brain tumor patients [19]. For example, phenformin, a mitochondrial complex I inhibitor, can increase the expression of miR-124, miR-137, and miR-let-7, leading to the inhibition of self-renewal of glioma stem cells [20]. Similarly, by targeting miR-324, a recent study has found that the inhibition of miR-324 confers protection against spontaneous seizures [21]. The present review would only focus on the advancement of miRNA researches in PTSD and TBI, and readers are referred to other comprehensive reviews for other diseases [9, 22, 23].

Although a certain number of miRNAs-focused studies have been conducted on PTSD and TBI, respectively, as the overlap between PTSD and TBI has raised mounting concerns, there is still a lack of studies dissecting the common miRNAs underlying both conditions and investigating their potential roles.

## Connections between TBI and PTSD

TBI is associated with a multitude of neuropsychiatric diseases owing to the convoluted pathophysiological changes that occur following the primary mechanical insults and the ensuing cascades of secondary injuries, causing neuron death, axon disruption, and neural circuits damage [24]. Aside from these cellular and histological changes following TBI, myriads of psychological changes have also happened, therefore spurring strong interests towards TBI in the field of psychiatry [25]. As is frequently accompanied by PTSD, TBI is also a common injury that contributes to the subsequent development of PTSD. Emerging evidence suggests that mild TBI (mTBI) renders the patients at higher risks of PTSD, while moderate-to-severe TBI exerts protective effects [7, 26]. The mTBI may increase the vulnerability to PTSD by inducing damage to the white matter structures that are involved in networks that negatively regulate the autonomic nervous system (ANS) activity, especially the uncinate fasciculus and the anterior limb of the internal capsule [27]. The abnormal ANS in PTSD patients can therefore elicit a cluster of hyperarousal symptoms, including sleep disturbance, exaggerated startle, hypervigilance, and explosive behaviors [28, 29]. Patients who sustained moderate to severe TBI can still develop PTSD, but less frequently. Such protective effects are possibly via disrupting traumatic memories formation, which underlies PTSD [30]. Compared with mTBI, moderate to severe TBI causes an overt structural brain injury, which leaves the patient with chronic functional and cognitive deficits and is associated with an increased risk for dementia such as Alzheimer’s disease (AD) later on in life [31, 32]. Generally speaking, PTSD and TBI share a breadth of overlapping neuropsychiatric symptoms and can be partially explained by the common underlying pathophysiological processes [33, 34].

**Fig. 3:**
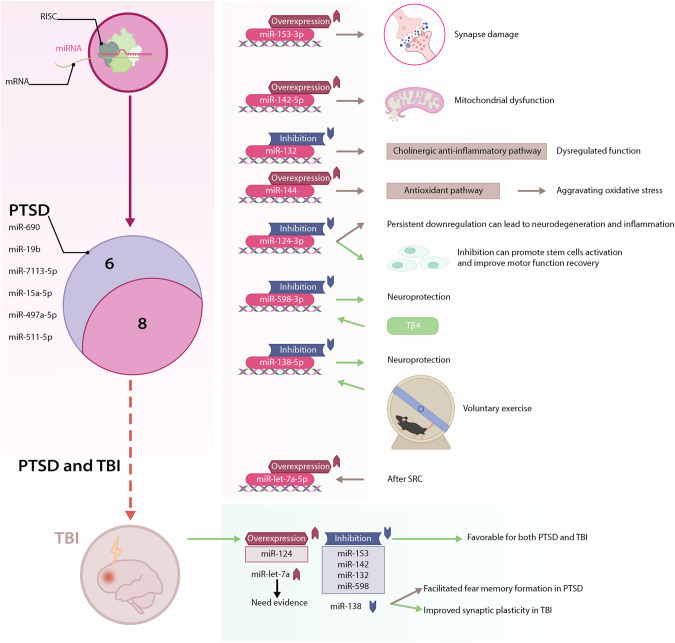
PTSD-TBI-common miRNAs reported in TBI. The eight PTSD-TBI-common miRNAs also play important roles in TBI. Overexpression of miR-153-3p, miR-142-5p, miR-144, and miR-let-7a-5p is reported in the pathological processes of TBI; inhibition of miR-598-3p, miR-138-5p shows neuroprotection effects. Moreover, inhibition of miR-132 is detrimental, and persistent downregulation of miR-124-3p can lead to neurodegeneration and inflammation, while evidence also suggests that inhibition of miR-124-3p promotes stem cell activation and motor function recovery.

In fact, miRNAs are critical regulators in many pathophysiological processes at the post-transcriptional level and were broadly investigated in both PTSD and TBI (See Tables 1–4**)**. Elucidating the role of miRNAs in PTSD and TBI, especially those PTSD-TBI-common miRNAs, whether as biomarkers or therapeutic targets, may provide new insights and hope for both conditions (See Figs. 1b, 3).

**Table 1:** Summary of miRNAs reported in recent PTSD preclinical studies.

Study	miRNA	Sample	PTSD model	Location	Findings in PTSD	Reported in TBI
2021 Park et al.	miR-690	mouse	Restraint stress	mPFC	miR-690 could prevent depressive-like behaviors and cognitive dysfunction following exposure to restraint stress	No
2022 Y.-L. Chen et al.	miR-153-3p	rat	SPS	hippocampus	miR-153-3p might alleviate PTSD-like behaviors by regulating cell morphology and reducing cell apoptosis in the hippocampus through mTOR-related signaling pathways	Yes 2014 Liu et al. 2019 A.M. Svingos et al.
2021 P.-Y. Nie et al.	miR-142-5p	rat	SPS	hippocampus	miR-142-5p downregulation could alleviate PTSD-like behaviors through attenuating neuroinflammation in the hippocampus by binding FMRP	Yes 2014 Sun et al. 2015 Wang et al. 2020 Schindler et al.
2020 L.-L. Ji et al.				amygdala	miR-142-5p may be an immediate response regulator of stress reactions and may participate in the pathophysiology of PTSD	
2020 P.-Y. Nie et al.	miR-132-3p	rat	SPS	hippocampus	miR-132-3p could modulate PTSD-like behaviors through FXR1 and FMRP regulation	Yes 2012 M. Valiyaveettil et al. 2013 Arai et al. 2019 Sessa et al.
2017 Murphy et al.	miR-144-3p	mouse	Fear conditioning and extinction	BLA	miR-144-3p plays a fundamental role in the rescue of impaired fear distinction	Yes 2014 Liu et al. 2016 Meissner et al.
2022 Chen et al.	miR-124-3p	rat	SPS	hippocampus	miR-124-3p might attenuate PTSD-like behaviors and neuroinflammation by downregulating TRAF6 in the hippocampus	Yes 2022 Zhao et al. 2022 Kang et al. 2018 N. Vuokila et al. 2015 W. Miao et al. 2019 Li et al. 2019 Sessa et al. 2019 Yang et al. 2020 Ge et al. 2020 N. Vuokila et al. (a) 2020 N. Vuokila et al. (b) 2017 Huang et al.
2018 Li et al.	miR-138-5p	mouse	CFC	dorsal hippocampus	Upregulation of miR-138-5p impaired the formation of fear memory	Yes 2015 W. Miao et al. 2015 Schober et al.
2021 O.M. Maurel et al.	miR-15a-5p miR-497a-5p miR-511-5p	mouse	AIS	hippocampus, hypothalamus, and mPFC	Expression of miR-15a-5p, miR-497a-5p, and miR-511-5p could discriminate susceptible and resilient mice	No
2019 Jones et al.	miR-598-3p	mouse	SEFL	BLA	miR-598-3p might mediate the susceptibility and resilience to stress enhancement of remote fear memory	Yes 2018 J. Osei et al.
2020 S.D. Linnstaedt et al.	miR-19b	human, rat	Not applicable; SPS and USS	human: blood; rat: amygdala, hippocampus, hypothalamus, adrenal glands, dorsal root ganglion, peripheral nerve, and blood	miR-19b might play a role in the development of PTWP and PTSS after exposure	No

**Table 2:** Summary of miRNAs reported in recent PTSD clinical studies.

Study	miRNA	Sample	PTSD model	Location	Findings in PTSD	Reported in TBI
2020 Bam et al.	miR-7113-5p	human	Not applicable	PBMCs	miR-7113-5p is decreased in PTSD patients and downregulated miR-7113-5p promotes inflammation by targeting the WNT signaling pathway	No
2022 Busbee et al.	miR-let-7a-5p	human	/	PBMCs	Downregulated miR-let-7a-5p promotes an inflammatory TH17 phenotype	Yes 2019 A.M. Svingos et al.
2020 S.D. Linnstaedt et al.	miR-19b	human, rat	Not applicable; SPS and USS	human: blood; rat: amygdala, hippocampus, hypothalamus, adrenal glands, dorsal root ganglion, peripheral nerve, and blood	miR-19b might play a role in the development of PTWP and PTSS after exposure	No

**Table 3:** Summary of PTSD-TBI-common miRNAs in TBI preclinical studies.

miRNA	TBI studies	Sample	Findings in TBI
miR-153	2014 Liu et al.	rat	miR-144, miR-153, and miR-340-5p may play important roles collaboratively in the pathogenesis of TBI-induced cognitive and memory impairments
miR-142	2014 Sun et al.	rat	The temporal expression pattern of miR-142-3p may be used as a molecular marker for TBI progression assessment
	2015 Wang et al.	rat	mitochondria-enriched miR-142-3p is decreased more than two-fold following TBI while levels in the cytosol is significantly increased
miR-132	2012 M. Valiyaveettil et al.	mouse	miR-132 is linked to cholinergic anti-inflammatory signaling after blast exposure
miR-144	2014 Liu et al.	rat	miR-144, miR-153, and miR-340-5p may play important roles collaboratively in the pathogenesis of TBI-induced cognitive and memory impairments
	2016 Meissner et al.	mouse	miR-144 is dramatically increased after TBI and may be central to the pathogenesis of trauma-induced brain damage
miR-124	2022 Zhao et al.	mouse	miR-124-3p attenuates brain microvascular endothelial cell injury in vitro by promoting autophagy
	2022 Kang et al.	rat	Downregulation of miR-124-3p could enhance BDNF downstream pathways and might lead to better outcomes in TBI
	2018 N. Vuokila et al.	rat, human	miR-124-3p is a chronic regulator of gene expression after TBI and is a potential treatment target
	2015 W. Miao et al.	mouse	Voluntary exercise prior to TBI leads to a high level of miR-124, which might improve the recovery
	2019 Li et al.	mouse	Increases in mir‑124‑3p in microglial exosomes confer neuroprotective effects by targeting FIP200‑mediated neuronal autophagy after TBI
	2019 Yang et al.	rat	miR-124 enriched exosomes promoted the M2 polarization of microglia and enhanced hippocampus neurogenesis after TBI by inhibiting the TLR4 pathway
	2020 Ge et al.	mouse	Increased microglial exosomal miR-124-3p alleviates neurodegeneration and improves cognitive outcome after rmTBI
	2020 N. Vuokila et al. (a)	rat	Persistent downregulation of miR-124-3p might contribute to post-injury neurodegeneration and inflammation
	2020 N. Vuokila et al. (b)	rat	Elevated acute plasma miR-124-3p level relates to the evolution of larger cortical lesion area after TBI
	2017 Huang et al.	mouse	Increased miR-124-3p in microglial exosomes following TBI inhibits neuronal inflammation and contributes to neurite outgrowth via their transfer into neurons
miR-598	2018 J. Osei et al.	rat	miR-598-3p is significantly increased in expression following TBI
miR-138	2015 W. Miao et al.	mouse	miR-138 was globally downregulated in TBI mice with voluntary exercise prior to impact and might exert a protective effect

**Table 4:** Summary of PTSD-TBI-common miRNAs in TBI clinical studies.

miRNA	TBI studies	Sample	Findings in TBI
miR-153	2019 A.M. Svingos et al.	human	miR-153-3p, miR-223-3p, and miR-let-7a-5p are significantly upregulated acutely following sport-related concussion
miR-142	2020 Schindler et al.	human	miR-142-3p expression is not detectable in the blood samples of any groups of patients within 6 h after the injury (severely injured patients without TBI, severely injured patients with TBI, patients with isolated TBI)
miR-124	2018 N. Vuokila et al.	rat, human	miR-124-3p is a chronic regulator of gene expression after TBI and is a potential treatment target
miR-138	2015 Schober et al.	human	The combination of miR-138/miR-744 levels completely discriminated patients who died from severe frontal cortex injuries or natural causes.
miR-let-7a	2019 A.M. Svingos et al.	human	miR-153-3p, miR-223-3p, and miR-let-7a-5p are significantly upregulated acutely following sport-related concussion

## Pathophysiological changes after PTSD and TBI and the roles of miRNAs

Both PTSD and TBI could result in brain structure disruptions; TBI is marked by primary injury caused by an external force, leading to focal or diffusive damages [35], while PTSD is associated with a reduced hippocampus size, possibly resulting from stress exposure [36]. Therefore, in TBI, the overlap in pathophysiology with PTSD is mostly in the secondary injury that follows the primary injury. Inflammation, mitochondrial dysfunction, excitotoxicity, calcium overload, and oxidative stress are common mechanisms that drive the secondary injury in TBI, ultimately disrupting the neural circuits and leaving patients with behavioral and cognitive impairments [37]. The pathophysiology of PTSD is characterized by neural circuits dysfunction, neurotransmitter dysregulation, and dysregulated stress response systems [38]. In addition to inflammation, excitotoxicity, and oxidative stress, which are considered the pathophysiological bases of PTSD and TBI [33], epigenetic alteration has been proposed as an important mechanism to better understand the pathophysiology of both PTSD and TBI [39, 40]. Typically, epigenetic regulations include DNA methylation, histone modification, chromatin remodeling, and miRNAs expression [41]. The miRNAs were widely studied and were believed to hold a key role in many pathophysiological conditions. Functioning as anti-inflammatory or pro-inflammatory factors, altered miRNAs expression in certain immune cells could regulate the inflammation at different stages via both positive and negative feedback [42]. It has also been established that miRNAs serve as a key role in maintaining the normal function and integrity of neural circuits in various aspects, including synaptic plasticity, CNS inflammation, axon outgrowth, and so on [9]. Upon stress, the miRNAs fine-tune the cells to adapt to the new environment by involving either negative or positive feedback loops [43]. For example, the HPA axis, which is designed to respond to stress, is suggestive of PTSD if dysfunctional. The miRNAs modulate the FK506-binding protein 5 (FKBP5), which is a critical modulator of the HPA axis, thereby regulating the stress response [39].

In this review, we summarized the recent advances in miRNA-related research in PTSD and organized them into four categories: inflammation-related miRNAs, neural circuits dysfunction-related miRNAs, stress response-related miRNAs, and sex-dependent miRNAs.

## Dysregulated miRNAs in PTSD

### Inflammation-related miRNAs in PTSD

#### miR-153

Chen et al. [44] found that downregulation of miR-153-3p in the hippocampus of rats could reduce apoptosis, increase neuronal dendritic spine density, and ultimately alleviate PTSD-like behaviors, possibly through the upregulation of sigma nonopioid intracellular receptor 1 (σ-1R) and PSD95 (postsynaptic density protein 95). As a transmembrane endoplasmic reticulum protein, σ-1R is widely distributed in the CNS, especially on neurons [45]. Activation of σ-1R has been reported to reduce memory impairment and anxiety-like behaviors induced by single-prolonged stress (SPS) through reversing the downregulated brain derived neurotrophic factor (BDNF) related signaling pathways [46]. Chen et al. showed that the σ-1R is involved in the mammalian target of rapamycin (mTOR) signaling and is a direct downstream target of miR-153. The mTOR signaling pathway might be involved in the regulation of σ-1R in response to SPS, and evidence suggested that σ-1R might also be an upstream regulator of the mTOR signaling pathway. However, PSD95, as a well-known synapse marker reflecting synaptogenesis or synaptic potentiation, is not predicted to be the target gene of miR-153, and the upregulation of PSD95 might be a downstream effect of σ-1R activation. Overall, inhibition of miR-153 could suppress inflammation via activation of σ-1R, which subsequently upregulates mTOR signaling, resulting in anti-inflammatory effects.

#### miR-124

miR-124 is probably one of the most thoroughly-studied miRNAs and is the most abundant miRNAs enriched within the brain. miR-124 affects a wide spectrum of pathological processes, including psychiatric disorders and CNS injuries. Chen et al. [47] found that the level of miR-124-3p was downregulated in the hippocampus of rats exposed to SPS, with the increased level of TNF receptor-associated factor 6 (TRAF6), which is a target gene of miR-124-3p. TRAF6 is an E3 ubiquitin ligase and serves as a signaling adaptor and scaffold for binding of both enzymes and target molecules, and it plays a pivotal role in regulating neuroinflammation via mediating the activation of the nuclear factor kappa-B (NF-κB) signaling [48, 49]. The overexpression of miR-124 could downregulate NF-κB activation by targeting TRAF6, thereby attenuating PTSD-like behaviors.

#### miR-7113

Bam et al. [50] analyzed peripheral blood mononuclear cells (PBMCs) from PTSD patients and controls and observed a significant upregulation of the Wnt/β-catenin signaling pathway in PTSD patients, leading to inflammation. Interestingly, miRNAs that were predicted to target these genes of the Wnt/β-catenin signaling pathway were downregulated, and miR-7113-5p was strongly predicted to target WNT10B. With mimic transfection assays, Bam et al. confirmed the regulatory role of miR-7113-5p to the Wnt/β-catenin signaling pathway by targeting WNT10B. Moreover, a more recent study has suggested that both the canonical Wnt/β-catenin and non-canonical Wnt/Ca^2+^ signaling pathways are involved in the medial prefrontal cortex (mPFC) of rats for fear memory retrieval using a contextual fear conditioning (CFC) paradigm [51].

#### miR-let-7a

Busbee et al. [52] reported miR-let-7a as the most significantly downregulated tumor protein (TP53) associated miRNA in PTSD PBMCs compared to controls and was shown to regulate CD4 + T helper-17 (Th17) cells negatively. The downregulation of TP53 and miR-let-7a could lead to abnormal inflammatory TH17 responses in PTSD. Additionally, a recent study has demonstrated that the T-lymphocyte-generated catecholamines are necessary for the abnormal inflammatory TH17 response in the repeated social defeat stress (RSDS) mice model [53]. Indeed, PTSD patients are predisposed to inflammatory and autoimmune diseases [54], and a randomized controlled trial found elevated levels of inflammatory cytokines even after one year in PTSD patients suffering from sexual assault, though with improved symptoms [55].

### Neural circuits dysfunction-related miRNAs

#### miR-142

L.-L. Ji et al. [56] found increased miR-142-5p levels in the amygdala of rats exposed to SPS. The inhibition of miR-142-5p exhibited increased Npas4 and BDNF expressions and decreased anxiety-like behaviors and memory deficits. The Npas4, as an activity-regulated transcription factor, is involved in mediating neuronal cell stress responses and is confirmed as the direct downstream target of miR-142-5p in this study. In fact, Npas4 has been reported that is highly expressed in the hippocampus CA3 region and may regulate a gene induction program that is essential for contextual fear memory [57]. Moreover, Npas4 can activate the transcription of BDNF in excitatory neurons to increase the number of inhibitory synapses on excitatory synapses [58]. BDNF is a well-known neurotrophin that has been implicated to be a potential risk factor for PTSD, unfortunately, our understanding of how BDNF modulates the risk of PTSD is still in its infancy [59].

Another study by P.-Y. Nie et al. [60] focused on the fragile X mental retardation protein (FMRP, also known as FMR1), a selective mRNA-binding protein that orchestrates neuronal development and synaptic plasticity [61], and the loss of FMRP could lead to deficits in trace fear memory [62]. Fragile X-related protein 1 and 2 (FXR1 and -2) are two paralogs of FMRP with overlap protein binding sites over 95%. Together, they compose the Fragile X family of RNA-binding proteins (FraX) [61, 63]. Not limited to the fragil X mental retardation syndrome (FXS) and autism, FraX is involved in arrays of psychiatric diseases, including anxiety, bipolar disorders, and schizophrenia [64]. P.-Y. Nie et al. found that the downregulation of miR-142-5p, which could bind to FMRP in the hippocampus of SPS-exposure rats, resulted in NF-κB signaling pathway downregulation and alleviated PTSD-like behaviors by attenuating the neuroinflammation and increasing levels of synaptic proteins including PSD95 and synapsin-I (SYN1). PSD95 and SYN1 are widely used post- and presynaptic markers, respectively, and their expression status could indicate the synapses number and function.

#### miR-132

Similarly, P.-Y. Nie et al. [65] explored another miR-132-3p and suggested that the FXR1 and FMRP are the downstream targets of miR-132-3p. The inhibition of miR-132-3p increased the expression of FXR1 and FMRP in the hippocampus of SPS-exposure rats and consequently alleviated PTSD-like behaviors, which may be attributed to the preserved levels of PSD95 and SYN1. The SYN1 level is related to the function of presynaptic structure, and the PSD95 is reflective of the postsynaptic structure function [66]. Interestingly, there are compelling data suggesting that the synaptic loss and deficits may compromise the neuroplasticity and impair resilience, leading to chronic symptoms and impaired treatment efficacy among PTSD patients [67]. Moreover, the FMRP expression was found essential for antidepressant-mediated synaptogenesis in a recent study [68]. Hence, the growing body of evidence supports the therapeutic potential of anti-miR-132 treatment via promoting synaptogenesis.

#### miR-144

Murphy et al. [69] revealed that the miR-144-3p overexpression in the basolateral amygdala (BLA) increased synaptic plasticity and rescued impaired fear extinction in mice model, possibly via the miR-144-3p-mediated regulation of plasticity-associated signaling cascades, including PI3K/AKT (active), MAPK/ERK (active), NOTCH (negative), and mTOR (negative) by targeting genes Pten, Spred1, Notch1, and Mtor. However, one recent study found that the enhanced PI3K/AKT signaling in the BLA could lead to persistent fear memory. Furthermore, the enhanced PI3K/AKT signaling also activates mTOR signaling, which ultimately drives fear memory formation [70]. Another cohort study identified the NOTCH signaling as an important mediator for PTSD risk after exposure to traumatic events using an integrated approach of genetic, epigenetic, and bioinformatics analyses [71].

#### miR-138

Li et al. [72] found a transient decrease of miR-138-5p in the dorsal hippocampus of CFC mice. In vivo/vitro evidence indicated that miR-138-5p downregulation facilitated the fear memory formation partly through the calpain 1 (Capn1)-mediated synaptic plasticity pathway by targeting Capn1. The Capn1 is a member of the calpain family of calcium-activated cysteine proteases and is widely expressed in the CNS. Activation of Capn1 has been implicated in long-term potentiation (LTP), which is generally accepted as one mechanism underlying memory formation. Moreover, hyperactivation of Capn1 is associated with Ca^2+^ overload following substantial physical trauma [73, 74].

### Stress response-related miRNAs in PTSD

#### miR-15a, miR-497a, and miR-511

Maurel et al. [75] evaluated the expression of FK506 binding protein 5 (FKBP5), BDNF, and a set of miRNAs targeting both FKBP5 and BDNF and observed lower transcript levels of miR-15a-5p, miR-497a-5p, and miR-511a-5p in the hippocampus and hypothalamus of PTSD-related susceptible mice, compared with resilient mice when subjected to the Arousal-Based Individual Screening (AIS), which is a novel PTSD animal model, and is specifically designed for trauma susceptibility and resilience research [76]. Only FKBP5 showed a significant negative correlation with the three miRNAs but not for BDNF. Moreover, FKBP5 was elevated after fear conditioning and even after fear extinction in the mPFC with respect to control and resilient mice and was supposed to be pathological [77]. However, Maurel et al. also reported that BDNF was increased in the mPFC of PTSD-related susceptible mice compared to others. Conversely, another study applying the single prolonged stress and electric foot shock (SPS&S) rat model has reported that the BDNF expression was decreased in mPFC [78]. In point of fact, many studies have found that the increased BDNF level could alleviate PTSD symptoms [79, 80], and plasma BDNF concentration was significantly reduced in veterans with PTSD [81]. In all, although the difference in PTSD animal models and sample size might influence the results, more studies are required to better our understanding of BDNF in PTSD.

#### miR-690

Analyzing RNA-seq data of the mPFC of Fkbp5 KO mice, Park et al. [82] found that miR-690 expression was significantly increased compared with wild-type control mice. Furthermore, the overexpression of miR-690 could prevent depressive-like behaviors and cognitive dysfunctions in the restraint stress mice model, suggesting its potential regulatory role in stress resilience [83]. FKBP5 is a stress-related gene that has been validated to be regulated by miRNAs. The deletion of FKBP5 can reduce depression-like phenotype and lead to stress resilience [11, 84]. Additionally, the mPFC, amygdala, and hippocampus are all consistently implicated in the occurrence of PTSD, and two subregions of mPFC were found that could control the fear and stress behaviors [24, 85].

### Sex-dependent miRNAs in PTSD

#### miR-19b

S.D. Linnstaedt et al. [86] investigated miR-19b in human cohorts and SPS and unpredictable sound stress (USS) rats. They found that miR-19b might be involved in the pathogenesis of post-traumatic widespread pain (PTWP) and post-traumatic stress symptoms (PTSS) by targeting circadian rhythm (CR) relevant genes in a sex-dependent manner. In accordance with human data, the miR-19b levels after trauma exposure were lower in female rats in blood and dorsal root ganglion (DRG) at the baseline. The miR-19b levels in blood samples of human cohorts were negatively correlated with the probability of developing PTWP and PTSS in female but positively for male patients. The converse outcomes between male and female patients might be explained by the estrogen-mediated miRNAs expressions. The estrogen stimulation using 17β-estradiol led to a decrease of miR-19b in female rats primary cultures of DRG neurons, which is consistent with a previous study [87]. In fact, the regulation of miRNAs expressions by estrogen has long been noticed and studied, and it is suggested that estrogen regulates miRNAs via both genomic and non-genomic mechanisms [88, 89]. Despite different traumatic events characteristics and environmental factors between female patients and male patients, the higher risk of developing PTSD for female patients could not be fully explained, and estrogen is considered a strong factor contributing to the phenomenon [90]. Lower estrogen levels at the time of trauma increase the vulnerability to PTSD in female patients, and the high levels of estrogen provide neuroprotective and anti-inflammatory effects against severe PTSD symptoms [91, 92]. Taken together, the accumulating evidence supports us to hypothesize that the important involvement of estrogen in the pathophysiology and treatment of PTSD could be partially through the common interactions with miRNAs.

#### miR-598

Interestingly, Jones et al. [93] reported another sex-dependent miRNA in stress-enhanced fear learning (SEFL) mice. The miR-598-3p was elevated in the BLA of male mice susceptible to SEFL but not in female mice susceptible to SEFL. Inhibition of miR-598-3p could not influence anxiety-like behaviors and was memory-specific and only to susceptible mice. The protective effect of miR-598-3p inhibition on SEFL was probably through the regulation of the actin cytoskeleton, which is required for learning and plasticity. The actin cytoskeleton pathway is intertwined with many cellular processes, such as motility, adhesion, and proliferation [94]. It is worth noting that the actin cytoskeleton has been a major focus of neural injury and regeneration research. The accumulation of actin at the wound site is essential for repair processes. For example, the actin cytoskeleton dictates the growth cone dynamics, which drives the neurite outgrowth [95, 96].

## The role of miRNAs in neuron injury and repair after TBI

The dysregulation of miRNAs has been widely documented in different phases of TBI [97, 98]. In the acute phase, miRNAs are involved in inflammation, apoptosis, and neuroprotection. For example, the reduced miR-5121 level secreted from microglial in the acute phase could suppress the neurite outgrowth and synapse formation by targeting the repulsive guidance molecule family member A (RGMa), which inhibit various neuroregeneration processes [99]. The increased levels of miR-155 levels in microglia or macrophages are proinflammatory and driving progressive inflammation via cytokines release and activation of neurotoxicity [100]. During the chronic phase, miRNAs can participate in the neuroreparation and neuroregeneration to promote the recovery and adaptation of the brain following injury. The miR-21 is chronically increased in the neuronal exosomes, and can inhibit the autophagy by targeting the Ras-related protein 11A (Rab11a), which is implicated in the autophagosomes formation [101]. The miR-124-3p is found persistently downregulated, which contributes to the chronic inflammation and neurodegeneration [102]. In summary, miRNAs are involved in the entire pathophysiological process of TBI, but the exact mechanisms still remain unclear, and comprehension of their expression patterns and versatile roles in TBI may provide valuable opportunities for future research.

## Common miRNAs shared by PTSD and TBI

### Inhibition of miR-153 is neuroprotective for PTSD and TBI

In TBI, the expression of miR-153 was increased by at least 1.8-fold in the ipsilateral hippocampus compared with sham control group and persisted for at least seven days after injury (peaked at 1 hour with near 6-fold overexpression). From 1 hour to 3 days after the controlled cortical impact (CCI) procedure, which is a common animal model of TBI, Liu et al. observed damaged synapse ultrastructure, including blurred synaptic cleft, thinner postsynaptic density (PSD) and shorter length synaptic active zone (SAZ) in the TBI groups [103]. Concordantly, A.M. Svingos et al. also reported significant upregulation of miR-153 in the blood of athletes following sport-related concussion (SRC) [104]. In PTSD, the miR-153 inhibition improved synapse plasticity and reduced apoptosis in the hippocampus, which might be related to the alleviation of PTSD-like behaviors. As the downstream target of miR-153, σ-1R is envisaged as a potential therapeutic target for TBI owing to its neuroprotective effects against endoplasmic reticulum stress, mitochondrial dysfunction, calcium overload, and excitotoxicity by its activation [105]. Hence, more studies are warranted to delineate the overlap synaptopathy of PTSD and TBI and further evaluate the neuroprotective effects of miR-153 for both PTSD and TBI.

### Anti-inflammation effect of miR-142 inhibition in PTSD is possibly through the mitochondrial protection

As is overexpressed in PTSD, the inhibition of miR-142 reduced neuroinflammation and alleviated PTSD-like behaviors. However, the expression pattern of miR-142 following TBI is more complex. Sun et al. examined miRNAs expressions at three time points after the CCI procedure: day1, day3, and day5 in the ipsilateral hippocampus of rats. The expression of miR-142 was slightly increased on day1 and day3 but surged to more than 2-fold compared with the sham control group on day5 [106]. Wang et al. suggested that the levels of miR-142 were increased in the cytosol but decreased in the mitochondria at 12 hours following CCI [107].

Nevertheless, Schindler et al. found the expression of miR-142-3p not detectable in the blood samples of any groups of TBI patients within 6 h after the injury [108], which might be explained by the expression trajectory of miR-142-3p post-TBI. Mitochondrial dysfunction is an intensively studied pathological feature in TBI and has been implicated in many psychiatric disorders, including PTSD [109]. The reduced ATP supply, increased reactive oxygen species (ROS) production, impaired Ca^2+^ buffering, and mitochondrial fission and fusion can induce PTSD symptoms, and related therapies might be helpful for PTSD [110]. Meanwhile, P.-Y. Nie et al. and L.-L. Ji et al. suggested that the downregulation of miR-142 resulted in altered NF-κB and BDNF signaling pathways, thus rendering protective effects. Besides, BDNF and NF-κB were also implicated in mitochondria function, and emerging evidence in neurodegenerative diseases revealed that mitochondrial dysfunction could promote neuroinflammation and vice versa [111, 112]. It is therefore intriguing to postulate that miR-142 could also medicate the mitochondrial dysfunction in PTSD patients.

### The alleviation of PTSD symptoms via miR-132 inhibition might be partially through cholinergic signaling modulation

M. Valiyaveettil et al. [113] analyzed miRNAs and cholinergic signaling pathways in the blast-induced TBI mice model. The expression of miR-132 was significantly reduced in the cerebellum, accompanied by dysregulated cholinergic anti-inflammatory signaling pathways. miR-132 is, in fact, reported in various CNS diseases and functions mainly in neural differentiation, neural growth and migration, and neural plasticity [114]. Of note, the silence of miR-132 in rat neural stem cells (eNSCs) induced upregulated expression of synaptic proteins, including PSD95 and SYN1 [115], which is consistent with what P.-Y. Nie et al. have discovered in PTSD patients. The stem cell therapies have been advocated as a potent approach to intervene in the course of both PTSD and TBI. Nevertheless, the possible tumorigenicity has impeded the clinical application [34]. In this case, miRNAs-based therapies could circumvent this problem as long as the critical miRNAs-mediated regulatory networks are identified and harnessed. Furthermore, cholinergic signaling is responsible for fear learning and extinction, and the augmentation of cholinergic signaling could boost fear extinction in SPS mice [116]. These lines of evidence suggest that miR-132 inhibition might also alleviate PTSD-like behaviors through cholinergic signaling modulation and is therefore worth further investigation.

### Inhibition of miR-144 is favorable for PTSD and TBI through the modulation of multiple signaling pathways

Murphy et al. reported that the overexpression of miR-144-3p in the BLA of impaired fear extinction mice could increase PI3K/AKT and MAPK/ERK activation while suppressing Notch and mTOR signalings and finally improve the synaptic plasticity and facilitate fear extinction. Additionally, both Liu et al. and Meissner et al. observed drastic upregulation of miR-144 in the acute phase of TBI and suggested that miR-144 overexpression might be involved in the pathogenesis of TBI via aggravating the oxidative stress by targeting antioxidant signaling pathways [103, 117]. After TBI, the PI3K level is decreased, whereas mTOR is activated, which ultimately results in autophagy [118, 119]. Nevertheless, other studies argue that the activation of mTOR signaling, in effect, promotes regeneration and recovery [119]. Noteworthy is that the pathological inflammation induced by upregulated miR-144 expression was also reported in bacterial infection [120], and even the downregulation of miR-144 was able to elicit proinflammatory cytokine production [121]. Moreover, a recent study applied subcutaneous administration of antago-miR-144-3p and reduced depression-related phenotype in stress-susceptible mice [122]. Taken together, the application of miR-144 inhibition to treat PTSD and TBI should be considered with prudence, and more evidence is needed to draw the final conclusion.

### Overexpression of miR-124 is favorable for PTSD and the non-acute phase of TBI

Similar to PTSD, the expression of miR-124 in the chronic phase of TBI is also downregulated, and the persistent downregulation of miR-124 might lead to neurodegeneration and inflammation [102, 123]. However, elevated plasma miR-124 level in the acute phase is associated with greater cortical lesion [124], and in line with this, Kang et al. found that miR-124 inhibition after TBI promoted subventricular zone (SVZ) neural stem cells (NSCs) activation and improved motor function recovery [125]. While other studies not in the acute phase of animal TBI model all suggested that the increased level of miR-124 is favorable against the injury [126–131]. In animal TBI models, stem cells originate from SVZ, migrate to the injury site and proliferate and generate neurons and glial cells to support neuroreparation and neuroregeneration in response to the injury [132]. Though less prevalent in the field of PTSD, stem cell therapy has been implied as a potential approach to PTSD treatment [133], and a recent study reported that the transplantation of induced pluripotent stem cell-derived neural progenitor cells (iPSC-NPCs) successfully promoted regeneration and motor function recovery in a rat PTSD model [134]. Still, future work is needed to chart the role of miR-124 as whether favorable or pathological in different phases of TBI, and findings with regard to miR-124 in PTSD should also be interpreted with caution.

### Actin cytoskeleton regulation via miR-598 inhibition might drive the neuroprotection effect of thymosin beta 4 in TBI

Jones et al. found that the inhibition of miR-598-3p improved structural plasticity in the BLA by regulating the actin cytoskeleton. Additionally, miR-598-3p is also overexpressed following TBI. J. Osei et al. treated rat TBI model with thymosin beta 4 (Tβ4), which has been implicated with neuroprotective potential and showed a significant reduction in miR-598-3p expression [135]. In fact, Tβ4 is encoded by the TMSB4X gene and is known as a G-actin binding protein that modulates the G-actin polymerization and is recently being identified with a potential anti-inflammatory role in the CNS [136]. Although TMSB4X is not predicted to be the target gene of miR-598-3p by TargetScan or miRBase database, these results suggest the neuroprotection effect demonstrated by Tβ4 in TBI might be partially ascribed to the miR-598-3p inhibition-mediated actin cytoskeleton regulation, and it remains to be determined if miR-598-3p could regulate the TMSB4X expression.

### Inhibition of miR-138-5p facilitates fear memory formation in PTSD but exerts protective effects in TBI possibly via increasing synapse plasticity

Interestingly, Li et al. found that the miR-138-5p downregulation facilitated fear memory formation partly by upregulating the Capn1-mediated synaptic plasticity pathway. Another study by W. Miao et al. observed global downregulation of miR-138 in the cerebral cortex of TBI mice with voluntary exercise prior to the impact compared with control groups and might play a protective role [127]. Hence, it is likely that the downregulation of miR-138 improved the synapse plasticity and finally led to better neurological function in the TBI mouse model. Additionally, Schober et al. reported that miR-138-5p is a candidate marker to assess TBI severity for forensic or therapeutic purposes [137].

### Overexpression of miR-let-7a in TBI might be compensatory and anti-inflammatory due to voluntary exercise

While downregulation of miR-let-7a-5p in PTSD could lead to abnormal inflammatory TH17 phenotype in PTSD. A.M. Svingos et al. found that expression of miR-let-7a-5p in blood was acutely upregulated following SRC compared to baseline levels among athletes [104]. Nevertheless, it remains to be examined whether miR-let-7a-5p is compensatory or proinflammatory, since W. Miao et al. suggested that the voluntary exercise prior to TBI could provide protection to some extent by upregulating certain miRNAs in mice [127], therefore, it is plausible to assume that the upregulation of miR-let-7a-5p in the athletes might actually be neuroprotective due to the routine training of athletes, and an additional control group should hopefully help explain the effect.

## Potential PTSD-TBI-common miRNAs for future research

**Fig. 4:**
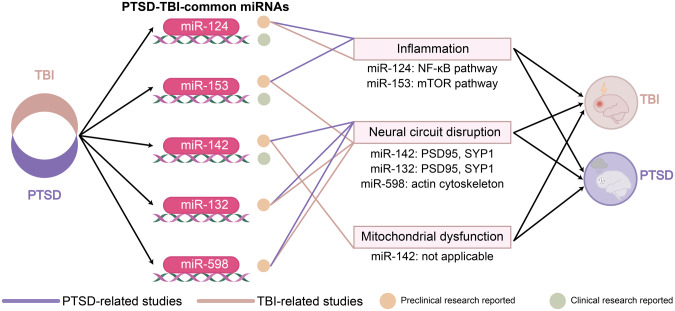
Potential PTSD-TBI-common miRNAs for future research. Five miRNAs (miR-124, miR-153, miR-142, miR-132, and miR-598) have been reported as potential targets that function in similar ways in PTSD and TBI, respectively. The expression changes of miR-124, miR-153, and miR-142 have already been reported in TBI clinical studies. Future research should investigate whether these miRNAs could provide new hope for PTSD and TBI through delicate modulation of inflammation and neural circuit function.

Our review highlights several miRNAs with promising therapeutic potential in PTSD and TBI, including miR-153, miR-142, miR-132, miR-598, and miR-124 (See Fig. 4). Studies on PTSD suggest that miR-153 and miR-124 may be used to regulate inflammation via the mTOR and the NF-κB pathway, respectively. The miR-142 and miR-132 may be utilized to preserve normal neural circuits function by modulating synaptic proteins (PSD95 and SYN1). The protective effects of miR-598 may be attributed to its regulation of the actin cytoskeleton pathways, and are sex-dependent. However, it is important to note that all the findings related to these five miRNAs were reported from preclinical animal studies of PTSD, and thus should be interpreted with caution. In the field of TBI, miR-153, miR-142, and miR-124 have been reported in clinical studies. However, most of these clinical studies only examined the expression status of miRNAs, without further investigating the underlying mechanisms. For future research focusing on miRNAs-based therapies for PTSD and TBI, it would be intriguing to see whether direct inhibition of the miR-153, miR-142, and miR-124 with antagomirs, or modulation of their target genes could provide benefits to the patients.

## Implications and future directions

The field of ncRNAs, including miRNAs, long non-coding RNAs (lncRNAs), and circular RNAs (circRNAs), is rapidly evolving and gaining significance. However, ncRNAs were considered junk and non-functional just several decades ago. Recently, the so-called “non-coding RNAs” are actually demonstrated with the capability of encoding proteins in some rare groups. Of note, circRNAs are probably the most promising ncRNAs for developing future novel treatments. Functioning as molecular sponges to bind miRNAs and proteins, scaffolds for the assembly of molecular components, or regulators for enhancing splicing and transcription, circRNAs can also be translated into proteins or peptides per se. The absence of 5’ N7-methylguanosine caps and 3’ polyadenylated tails endows circRNAs way more stability (resistant to canonical RNA decay pathways) compared with other linear ncRNAs [138], which makes them unexceptional vectors for drug delivery. For more about circRNAs, readers are referred to another elaborate review [139]. Unfortunately, currently available studies investigating circRNA-miRNA-mRNA regulatory axis in both areas of PTSD and TBI are scarce and warranted to fully utilize the miRNAs as the therapeutic targets.

It is also essential to note that the discovery of biomarkers for PTSD and TBI remains an open issue as most of the proposed biomarkers are correlational and offer limited mechanistic understanding. The emerging miRNAs-based biomarkers hold great promise for diagnosis and treatment. The brain-derived extracellular vesicle (EV) miRNAs in the peripheral circulation blood have been suggested as one of the best sources of miRNAs related to the PTSD and TBI, because the EV-packaged miRNAs are blood-brain barrier (BBB) permeable, degradation-stable, and furthermore, specific to many neuropsychiatric diseases [140]. A recent study evaluated the levels of EV proteins and miRNAs in the peripheral blood from a cohort of 144 military personnel, further solidifying the role of miRNAs in peripheral circulation blood as biomarkers for PTSD and TBI [141]. Moreover, miRNAs-based biomarkers can offer more mechanistic insights due to their epigenetic modulation ability, thereby providing a clearer link to the underlying signaling pathways implicated in PTSD and TBI.

Furthermore, the spatiotemporal expression pattern and tissue specificity of miRNAs and other ncRNAs should be appreciated. Indeed, several brain regions: the orbitofrontal cortex, the dorsolateral prefrontal cortex, and the hippocampus are vulnerable to both PTSD and TBI [24]. Next, it would be interesting to explore and compare the spatiotemporal expression pattern of miRNAs in these brain regions, which might hopefully help illuminate the regulatory role of miRNAs involved in these common pathophysiological processes between PTSD and TBI. The spatiotemporal sequencing technique is a new approach to analyze the expression status of genes or transcripts at a cellular resolution. It was already applied to determine the expression pattern of miRNAs during the CNS development of mice [142]. Unfortunately, similar studies on PTSD or TBI are rather limited.

Meanwhile, both symptoms of PTSD and TBI can vary upon time. PTSD symptoms gradually fade away but can be evoked given exposure to certain events and depend on the resilience of the patient, which is a dynamic process that has recently been found to be associated with the expression of specific miRNAs in certain brain hippocampal regions [143]. Emerging evidence has suggested that miRNAs could be highly involved in the resilience process [144, 145]. However, their regulatory roles in the resilience process are currently less studied and worth future investigations. One important reason is that most PTSD animal models focus mainly on the traumatic exposure while overlooking the contributions of other risk factors and their dynamic changes [146]. In TBI, things become trickier by virtue of biological processes that play a double-edged role in response to injury, they might help protect salvageable neurons and contain inflammation in the acute phase; however, they impede neuroregeneration and neurorestoration in the chronic phase. Thus, as key regulators driving these dynamic pathophysiological changes, miRNAs may be used as potential biomarkers and therapeutic targets for PTSD and TBI.

Additionally, sex differences should also be bear in mind when evaluating the efficacy of miRNA treatment, as the miR-19b and miR-598-3p were both reported with sex-dependency among PTSD studies. As a matter of fact, sex steroid hormones, including estrogen and testosterone, have been reported to have a significant influence on miRNAs regulation [147], and even more, the epigenetic modulation [148].

## Concluding remarks

The links between PTSD and TBI are accumulating and can mutually benefit related studies concerning both diseases. Nevertheless, this overlap has spawned even more questions, and how to identify and disentangle the intricate gene regulatory networks under the hood from similar pathophysiological processes is crucial to enable effective treatments in the future. Till now, our knowledge of miRNAs is pretty mature, and the development and application of miRNAs-based therapies are making steady progress. Therefore, it makes sense to believe that, whether as biomarkers for distinguishing PTSD status and monitoring TBI progression or as therapeutic targets for drug intervention, considering the nature of targeting multiple genes and the critical regulatory role in implicated signaling pathways, miRNAs indisputably bring new insights and opportunities to novel future therapies and more studies are therefore advocated to pave the way to the final solution.
